# Best practices in assessing cardiac protein *O*-GlcNAcylation by immunoblot

**DOI:** 10.1152/ajpheart.00104.2023

**Published:** 2023-08-04

**Authors:** Luyun Zou, Dingguo Zhang, Chae-Myeong Ha, Adam R. Wende, John C. Chatham

**Affiliations:** Division of Molecular and Cellular Pathology, Department of Pathology, University of Alabama at Birmingham, Birmingham, Alabama, United States

**Keywords:** cardiac, heart, immunoblot, O-GlcNAc

## Abstract

The modification of serine and threonine amino acids of proteins by *O*-linked *N*-acetylglucosamine (*O*-GlcNAc) regulates the activity, stability, function, and subcellular localization of proteins. Dysregulation of *O*-GlcNAc homeostasis is well established as a hallmark of various cardiac diseases, including cardiac hypertrophy, heart failure, complications associated with diabetes, and responses to acute injuries such as oxidative stress and ischemia-reperfusion. Given the limited availability of site-specific *O*-GlcNAc antibodies, studies of changes in *O*-GlcNAcylation in the heart frequently use pan-*O*-GlcNAc antibodies for semiquantitative evaluation of overall *O*-GlcNAc levels. However, there is a high degree of variability in many published cardiac *O*-GlcNAc blots. For example, many blots often have regions that lack *O*-GlcNAc positive staining of proteins either below 50 or above 100 kDa. In some *O*-GlcNAc blots, only a few protein bands are detected, while in others, intense bands around 75 kDa dominate the gel due to nonspecific IgM band staining, making it difficult to visualize less intense bands. Therefore, the goal of this study was to develop a modifiable protocol that optimizes *O*-GlcNAc positive banding of proteins in cardiac tissue extracts. We showed that *O*-GlcNAc blots using CTD110.6 antibody of proteins ranging from <30 to ∼450 kDa could be obtained while also limiting nonspecific staining. We also show that some myofilament proteins are recognized by the CTD110.6 antibody. Therefore, by protocol optimization using the widely available CTD110.6 antibody, we found that it is possible to obtain pan-*O*-GlcNAc blots of cardiac tissue, which minimizes common limitations associated with this technique.

**NEW & NOTEWORTHY** The post-translational modification of proteins by *O*-linked *N*-acetylglucosamine (*O*-GlcNAc) is recognized as mediating cardiac pathophysiology. However, there is considerable variability in the quality of *O*-GlcNAc immunoblots used to evaluate changes in cardiac *O*-GlcNAc levels. Here we show that with relatively minor changes to a commonly used protocol it is possible to minimize the intensity of nonspecific bands while also reproducibly generating *O*-GlcNAc immunoblots covering a range of molecular weights from <30 to ∼450 kDa.

## INTRODUCTION

The modification of serine and threonine amino acids of proteins by *O*-linked *N*-acetylglucosamine (*O*-GlcNAc) was first reported in 1984 (1). In contrast to traditional *N*- and *O*-linked glycosylation, *O*-GlcNAc is a rapidly reversible modification regulated by *O*-GlcNAc transferase (OGT), which catalyzes its attachment to proteins and *O*-GlcNAcase (OGA), which removes the modification (2). Its reversible nature and its modification of serine and threonine amino acids have drawn analogies between *O*-GlcNAcylation and phosphorylation (3). Like phosphorylation, *O*-GlcNAcylation can regulate the activity, stability, and subcellular localization of proteins (2).

Around 2001, it was recognized that cellular *O*-GlcNAc levels increased in response to high glucose levels, raising a possible link between increased *O*-GlcNAcylation and glucose toxicity (4–7). These and other studies demonstrated that under conditions of nutrient excess, as seen in diabetes, there was a sustained increase in cellular *O*-GlcNAc levels that potentially contributed to insulin resistance (8). Increased *O*-GlcNAc levels were also linked to the complications associated with diabetes, including the adverse effects of diabetes on the heart and vascular system (5, 9–13). It is now clear that *O*-GlcNAc levels in the heart are also increased in the absence of systemic metabolic diseases such as cardiac hypertrophy (14–17), heart failure (14, 18–20), and normal aging (21, 22). In addition, acutely increasing cardiac *O*-GlcNAc levels has been shown to be cardioprotective (23–26), and decreasing *O*-GlcNAc levels increases cardiomyocyte injury in response to oxidative stress (27). As a result, there is a growing interest in determining the role of *O*-GlcNAcylation in mediating cardiac pathophysiology and the potential for targeting it as a therapeutic intervention.

Unlike phosphorylation, commercially available site-specific *O*-GlcNAc antibodies are currently limited to only a few proteins such as those available from GlycoScientific (Athens, GA). A few site-specific *O*-GlcNAc antibodies have been raised against specific protein sites, such as c-Myc (28), tau (29, 30), sirtuin 1 (SIRT1) (31), and collapsin response mediator protein-2 (CRPM2) (32), but are not commercially available. Consequently, given the limited number and availability of site-specific *O*-GlcNAc antibodies, studies of *O*-GlcNAcylation in the heart, as well as other tissues, typically use pan-*O*-GlcNAc antibodies for semiquantitative evaluation of overall *O*-GlcNAc levels, rather than determining changes in *O*-GlcNAc levels of specific proteins. There are non-antibody-based approaches, such as the Click-iT *O*-GlcNAc Enzymatic Labeling System, available from several vendors; however, these methods involve additional steps, including protein precipitation and as a result, are more time-consuming. In addition, depending on the tissue lysis protocol, cell surface glycoproteins can also be labeled using these techniques and require treatment with PNGase F to remove them before enzymatic labeling (33). Therefore, because of the limited number of sample processing steps and the ease of use, pan-*O*-GlcNAc antibodies remain widely used to evaluate *O*-GlcNAc levels in cardiac and other tissues.

Thousands of proteins have been identified as *O*-GlcNAc targets (34) covering a wide range of molecular weights; however, there is considerable variability in published cardiac *O*-GlcNAc blots, often lacking *O*-GlcNAc positive staining of proteins either below 50 or above 100 kDa (11, 24, 35), whereas others exhibit a broader range of molecular weight bands (36). In some *O*-GlcNAc blots, only a few protein bands are detected and in others intense bands around 75 kDa dominate the gel, making it difficult to visualize and quantify less intense bands. Such variability makes it very difficult to compare results from different studies. Therefore, the goal of this study was to develop a protocol where *O*-GlcNAc levels of proteins ranging from ∼30 to 250 kDa could be routinely detected in cardiac tissue extracts using the widely commercially available *O*-GlcNAc antibody, CTD110.6.

## METHODS

### Animal Experiments

All experimental protocols using mice in this study were approved by the University of Alabama at Birmingham (UAB) Institutional Animal Care and Use Committee (IACUC) and adhered to the National Institutes of Health’s *Guide for the Care and Use of Laboratory Animals* (NIH Publication No. 85-23, Revised 1996). All animals received standard chow and water on an ad libitum basis, and lighting was maintained on a 12-h:12-h light/dark cycle. As this study was focused on protocol optimization, sex, age, or strain were not examined. Male C57BL/6J mice, 6–14 wk of age, were used throughout. In all studies, there were a minimum of two biological replicates and two technical replicates.

### Antibodies

Details of all the antibodies used in this study are listed in Table 1.

**Table 1.** List of all antibodies used in this study including vendors, catalog numbers, dilutions, and concentrations

Antibody	Source	Catalog No.	Dilution	Concentration, µg/mL#
*O*-GlcNAc antibodies				
CTD110.6	UAB Core*	N/A	1:2,000	N/A
CTD110.6	Cell Signaling	9875	1:1,000	3,160
CTD110.6	Sigma-Aldrich	MABS 1254	1:2,000	500
CTD110.6	Sigma-Aldrich	O7764	1:2,000	2,000
RL2	Sigma-Aldrich	MABS 157	1:1,000	500
RL2	Invitrogen	MA1-072	1:2,000	2,000
HGAC85	Novus Biologicals	NB300-614	1:1,000	N/A
MultiMab rabbit mAb mix	Cell Signaling	82332	1:1,000	1,878
Other antibodies				
GAPDH	Abcam	Ab8245	1:20,000	2,000
cTnI	Cell Signaling	4002	1:1,000	30
Tubulin	Applied Biological Materials	G098	1:3,000	1,000
Lamin B1	Abcam	ab133741	1:3,000	295
MYPT	Abcam	Ab32519	1:2,000	704
α1NKA	MilliporeSigma	16-243-MI	1:1,000	500
*Secondary antibodies*				
HRP anti-mouse IgM	Santa Cruz	sc2064*	1:4,000	400
Anti-mouse IgG	Bio-Rad	170-6,516	1:2,000	900
Anti-rabbit IgG	Bio-Rad	170-6,515	1:2,000	900
Goat anti-mouse IgM	Invitrogen	31,440	1:4,000	800
Goat anti-mouse IgM H&L Chain Specific Peroxidase Conjugate	Calbiochem	401,225	1:4,000	1,000
Goat anti-mouse IgM heavy chain secondary antibody [HRP] (pre-adsorbed)	Novus Biologicals	NBP1-75193	1:4,000	900

*No longer available. #Concentrations of antibodies can vary between different lots. The ones listed here are from the lots used in these experiments, which may be different from those obtained at different times. HRP, horseradish peroxidase; N/A, not available.

### Reagents

Details of all the reagents used in this study are listed in detail in Table 2.

**Table 2.** List of all reagents used in this study with vendors, catalog numbers, and additional characteristics

Reagent	Source	Catalog No.	Characteristics
2-Mercaptoethanol	Bio-Rad	161-0710	≥98% pure 2-mercaptoethanol (14.2 M)
Bromophenol blue	Sigma	B5525	
BSA	Sigma	A7906	pH 7, ≥98%
Digitonin	Calbiochem	300410	
DTT	Fisher	BP172-25	>99%
EDTA	Sigma	E9884	ACS reagent, 99.4–100.6%
EGTA	Sigma	E4378	≥97.0%
Glycerol	Sigma	G5516	For molecular biology, ≥99.0%
GlcNAc	Sigma	A8625	≥99%
HEPES	Sigma	H3375	≥99.5% (titration)
KCl	Sigma	P5405	≥99.0%
Methanol	Fisher	A412P-4	99.8%
MgCl_2_	Sigma	M8266	anhydrous, ≥98%
Na_3_VO_4_	Aldrich	450243	99.98% trace metals basis
NaF	Sigma	S7920	BioXtra, ≥99%
NaCl	Sigma	S7653	BioXtra, ≥99.5% (AT)
NP-40	Fluke	74385	
Na-deoxycholate	Fisher	BP349-100	≥99%
PBS 1×/1% casein	Bio-Rad	161-0783	
Protease inhibitor cocktail	Sigma	P8340	
SDS	Bio-Rad	161-0302	
Tris–HCl	Sigma	T3253	≥99.0% (titration)
Triton X-100	Sigma	T8787	
Tween	Bio-Rad	170-6531	100% Nonionic detergent

### Heart Isolation and Tissue Lysis

Following cervical dislocation, the left and right ventricles were rapidly removed and placed in ice-cold saline. The heart was allowed to beat two to three times to expel blood and then cut into three smaller pieces and placed in a precooled collection tube and stored in liquid nitrogen. Subsequently, 20–50 mg of powdered heart tissue was added to ∼600 µL of either a mild detergent or RIPA lysis buffer.

#### Mild detergent lysis buffer.

Mild detergent lysis buffer (MDB) consisted of 20 mM HEPES, 1.5 mM MgCl_2_, 20 mM KCl, 20% glycerol, 0.2 mM EDTA, 1% Triton X-100, 2 mM Na_3_VO_4_, 10 mM NaF, and fresh 2% protease inhibitor cocktail, which inhibits serine, cysteine, acid proteases, and aminopeptidases (Sigma P8340).

#### RIPA lysis buffer.

Radioimmunoprecipitation assay (RIPA) lysis buffer consisted of 50 mM Tris, 0.5% Na-deoxycholate, 0.1% SDS, 150 mM NaCl, 25 mM NaF, 1 mM EDTA, 1% NP-40, and fresh 2% protease inhibitor cocktail (Sigma P8340).

#### Tissue lysis protocol.

Each tissue sample was homogenized in a 5-mL tube using a PowerGen 125 homogenizer (Fisher Scientific) at ∼25,000 rpm and transferred to a labeled Eppendorf tube on ice. Samples were allowed to sit on ice for 20–30 min and vortexed at 5-min intervals. Finally, the samples were centrifuged at 4°C for 20 min at 13,400 rpm. We emphasize consistency in the homogenization process, which we believe contributes to greater reproducibility in subsequent immunoblots; however, we have not explicitly compared the effects of different durations of homogenization and vortexing on subsequent *O*-GlcNAc immunoblots.

As soon as possible following lysis, protein concentrations were determined, lysates were reduced in ×6 sample loading buffer, consisting of 0.5 M Tris–HCl (pH 6.8), 10% SDS, 30% glycerol, 6% 2-mercaptoethanol, and 0.012% bromophenol blue, and heated to 95°C for 5 min, before storing at −80°C until subsequent analyses. Temperature and heating duration were not further tested in the current optimization. In much earlier studies, we found that minimizing the time the lysate is stored before being placed in a sample loading buffer improves immunoblot quality and reproducibility, however, we did not evaluate this further. We therefore try to complete lysate isolation from tissue to reduction in 1 day. If repeated, *O*-GlcNAc measurements are to be performed, it is recommended that samples are aliquoted before −80°C storage to avoid freeze thaws, as we observed in earlier studies that repeated freeze thaws resulted in a gradual loss in *O*-GlcNAc intensity. These experiments were not repeated for this study.

### Cell Fractionation Methods

#### Myofilament fractionation.

Lysed 20–50 mg of powdered mouse heart tissue as aforementioned, using either MDB or RIPA lysis buffer. We also tested the lysis buffer described by Yin et al. (37), specifically for the isolation of myofilament proteins, which consisted of 50 mM Tris–HCl, 5 mM EGTA, 2 mM EDTA, 5 mM DTT, 0.05% digitonin, and fresh 2% protease inhibitor cocktail.

Myofilament protein isolation was performed with slight modifications as described by Yin et al. (37). Following lysis, samples were put on ice for 20–30 min and vortexed every 5 min. The samples were then centrifuged at 4°C for 30 min at 14,000 rpm (Eppendorf microcentrifuge 5415 C/5415R), and the supernatant that consists of the cytosolic fraction was transferred to a precooled microcentrifuge tube. The pellet was dissolved in the lysis buffers aforementioned with the addition of 1% Triton X-100 and centrifuged for 30 min at 14,000 rpm at 4°C. The supernatant which contains the membrane fraction was transferred to a precooled microcentrifuge tube. The remaining pellet contains the myofilament fraction. All three fractions were reduced in equal volumes of Laemmli sample buffer. The protocol of Yin et al. (37) included 5 mM DTT in the Laemmli sample buffer. Therefore, 2-mercaptoethanol was added to samples that used MDB or RIPA lysis buffers. All samples were boiled for 5 min and kept at −80°C until subsequent analyses.

#### Nuclear and cytoplasmic fractionation.

Tissue lysis and fractionation were performed using the NE-PER Nuclear and Cytoplasmic Extraction Kit from Thermo Fisher Scientific (Cat. No. 78833), with a few modifications. Twenty milligram of powdered heart tissue was placed in a microcentrifuge tube and washed with PBS one time, before centrifugation at 3,000 rpm for 5 min. Supernatant was removed and discarded, and the remaining heart tissue was suspended in 200 mL of cytoplasmic extraction reagent I (CER I) and incubated on ice for 10 min, followed by the addition of 11 µL of CER II, vortexing for 5 s and incubation on ice for 1 min. Following additional vortexing for 5 s, the samples were centrifuged at 13,400 rpm for 5 min. The supernatant, which is the cytoplasmic fraction, was transferred to a prechilled microcentrifuge tube. The remaining insoluble pellet was suspended in 100 µL of ice-cold nuclear extraction reagent (NER) and vortexed at high speed for 25 s. This was repeated every 5 min for a total of 60 min followed by centrifugation at 13,400 rpm for 15 min. The supernatant, which is the nuclear fraction, was transferred to a prechilled tube. All samples were stored at −80°C until subsequent analyses.

### Western Blot Analysis

Following the thawing of samples, ∼25 µg of protein/lane were loaded onto 8% gels unless stated otherwise and separated by SDS-PAGE. In much earlier studies, we had determined that this protein concentration was sufficient to obtain reproducible *O*-GlcNAc blots using the CTD110.6 antibody; therefore, we did not use other protein concentrations. Nevertheless, as recommended by Fahie et al. (38), a dilution series is important when establishing a new protocol. A transfer buffer, consisting of 25 mM Tris and 192 mM glycine (pH 8.3) and containing 10% methanol with 0-0.05% SDS was used to transfer proteins to 0.45-µm pore size, Immobilon-P PVDF membrane (Millipore). In earlier studies, we found that PVDF performed better than nitrocellulose membranes (data not shown) and therefore chose not to compare the different membranes here. Immunoblotting was performed using a rapid immunodetection method for Immobilon-P (Millipore Technical Note TN051). Unless stated otherwise, the transfer voltage was 100 V, for 70 min at room temperature. We also tested 70 V for 3 h at room temperature, and 25 V at 4°C overnight and concluded that 100 V for 70 min at room temperature provided the optimal results (Supplemental Fig. S1; note: all Supplemental material is available at https://doi.org/10.6084/m9.figshare.23589084). Except where stated otherwise, all *O*-GlcNAc blots used the UAB CTD110.6 antibody.

Immunoblotting was performed with 1:2,000 anti-*O*-GlcNAc antibody CTD110.6, using either 1% casein-phosphate-buffered saline (PBS) or 3% bovine serum albumin (BSA)/Tris-buffered saline-0.1% Tween (TBST) overnight at 4°C. The casein-PBS membranes were equilibrated in 100% methanol and air-dried. The dried membrane was then rehydrated before immunoblotting. Membranes were washed three times with either PBS or TBST before immunoblotting with CTD110.6. To determine the specificity of the antibody for *O*-GlcNAc, immunoblotting was performed in the presence of 10 or 100 mM GlcNAc as described in detail elsewhere (38).

The membranes were incubated with the appropriate horseradish peroxidase (HRP)-conjugated secondary antibodies for 1 h at room temperature or 2 h at 4°C. After further washing three times for 15 min each, in TBST or PBS as appropriate, the immunoblots were developed with enhanced chemiluminescence (PerkinElmer Life Sciences) digitally using the Amersham Imager 600.

## RESULTS

We focused on the CTD110.6 anti-*O*-GlcNAc antibody because it remains one of the most widely used pan-*O*-GlcNAc antibodies and has also been the mainstay of the work in our own laboratories for many years. However, we believe that the principles outlined here are broadly applicable to other pan-*O*-GlcNAc antibodies. We focused on three specific aspects of sample preparation and immunoblotting: *1*) lysis buffer, *2*) blocking agent, and *3*) transfer buffer.

### Lysis Buffers and Blocking Agents

For the lysis buffer, we compared a MDB with RIPA, a stringent denaturing buffer, both of which are routinely used in our laboratories.

Frequently used blocking agents are nonfat dried milk, casein, and BSA; although, other commercial agents are available such as EveryBlot from Bio-Rad (Cat. No. 12010947) and BLOTTO from Thermo Fisher Scientific (Cat. No. 37530). In much earlier studies, we found that in our hands, dried skim milk did not work well as a blocking agent for CTD110.6; therefore, we did not repeat these experiments here. We did not test commercial blocking agents because of the proprietary nature of their composition. Therefore, we compared casein, which has long been used in the Chatham laboratory with BSA, which has been in use in the Wende laboratory. As aforementioned, casein was used with dried membranes, whereas BSA used the more commonly used wet membranes. These conditions had been optimized in earlier studies for each of the blocking agents and because of our initial results, we decided not to directly compare dry versus wet membranes with different blocking agents.

**Figure 1.**
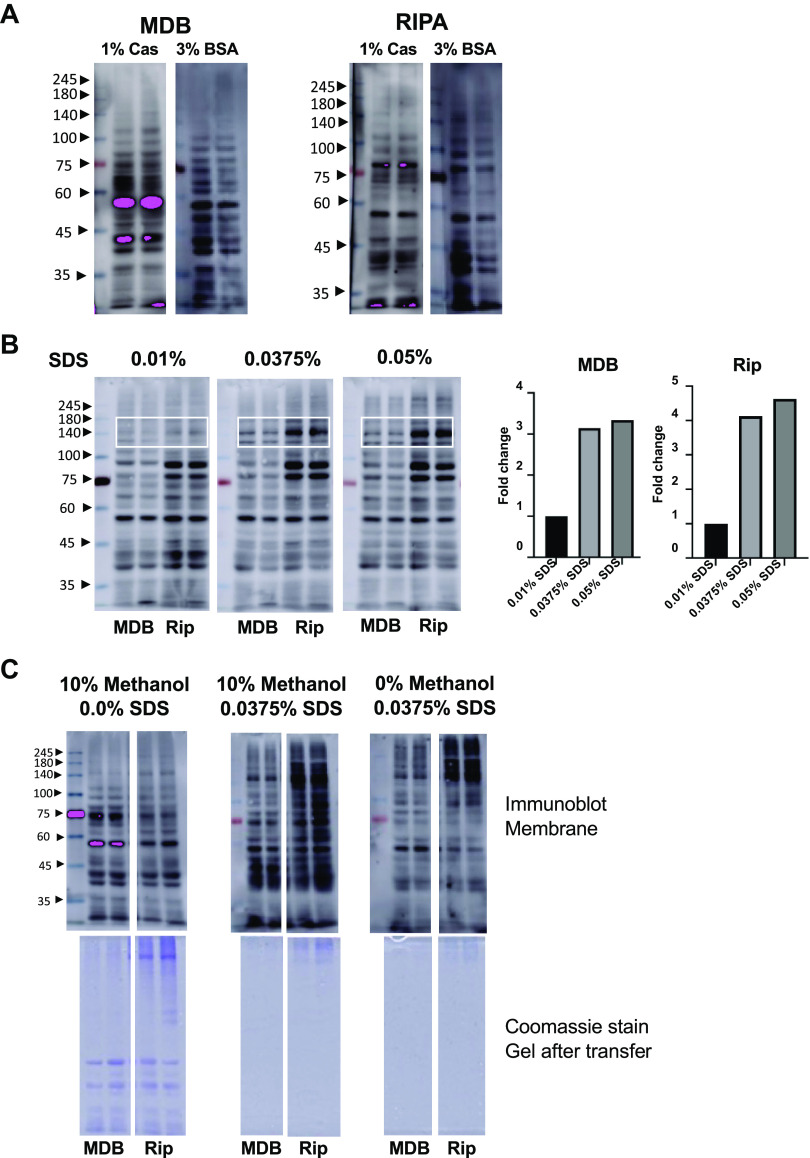
*A*: *O*-linked *N*-acetylglucosamine (*O*-GlcNAc) immunoblots of cardiac tissue extracted using either mild detergent buffer (MDB) or radioimmunoprecipitation assay (RIPA, Rip) lysis buffer, with either 1% casein (Cas) or 3% bovine serum albumin (BSA) as blocking agent. Note that the MDB and RIPA samples were run on the same gels but were separated to compare the different blocking agents with each lysis buffer. *B*, *left*: effects of adding 0.01–0.05% sodium dodecyl sulfate (SDS) to transfer buffer with 10% methanol with 3% BSA as blocking agent in cardiac extracts using either MDB or RIPA lysis buffer. *B*, *right*: densitometry analysis of the area indicated by the white box for both MDB and RIPA blots. As we were comparing differences between transfer buffer composition, the gels were by necessity run separately. However, for image analysis purposes, the gels were merged into a single image; see text and Supplemental Fig. S6*A* for more details on the analysis. *C*, *top*: *O*-GlcNAc immunoblots comparing the effects of 10 vs. 0% methanol and 0 or 0.375% SDS in transfer buffers. *C*, *bottom*: Coomassie stain of gel following transfer for the immunoblots shown above. The focus was on comparisons of SDS concentrations and methanol on molecular weight range characteristics. Therefore, the MDB and RIPA samples were run on the same gels but were separated to compare the different SDS concentrations with each lysis buffer. Note that some of the blots shown here were cropped for ease of presentation. Uncropped blots are shown in Supplemental Fig. S2.

*O*-GlcNAc blots in samples isolated with MDB or RIPA buffer, using either 1% casein or 3% BSA in the blocking buffer are shown in Fig. 1*A.* Although the differences are relatively minor, we concluded that 3% BSA resulted in a slightly sharper banding pattern compared with casein regardless of the lysis buffer used; however, this is a subjective evaluation and could vary depending on other conditions. Regardless of the blocking agent, very few if any *O*-GlcNAc positive bands were observed >100 kDa, a common limitation in our own studies (11, 24, 35), as well as others. Thus, we concluded that the different lysis buffers and blocking agents did not have a major effect on the molecular weight range of *O*-GlcNAc positive bands detected. Note that comparisons between MDB and RIPA lysis buffer were used solely for molecular weight range characteristics and not for differences in the banding pattern, because the populations of proteins isolated with these two buffers will be distinct.

### Transfer Buffers

In transfer buffers, 10–20% methanol is typically used to prevent gel swelling during transfer and improve the efficiency of protein adsorption onto the membrane. However, methanol can potentially adversely affect transfer efficiency because of changes in gel pore size and protein precipitation, among other factors (39). SDS is usually not included in the transfer buffer because the negative charge imparted to proteins can cause them to pass through the membrane. On the other hand, low concentrations of SDS can be useful for proteins that tend to precipitate and also help the transfer of high molecular weight proteins (39). Therefore, we tested transfer buffers that included SDS (0–0.05%) in the presence and absence of 10% methanol. The addition of SDS to the transfer buffer resulted in a little foaming, which interfered with the transfer process; however, this was minimized by making the transfer buffers ∼24 h before use.

In Fig. 1*B*, we show the effect of adding 0.01–0.05% SDS in the transfer buffer containing 10% methanol and 3% BSA as the blocking agent. Regardless of the lysis buffer, the addition of 0.0375 or 0.05% SDS resulted in more *O*-GlcNAc positive bands over a wider range of molecular weights. Densitometric analyses demonstrate the marked increase in the intensity of proteins between ∼140 and 180 kDa with the addition of SDS to the transfer buffer (see *Densitometric Analysis of O-GlcNAc Blots* and Supplemental Fig. S6*A* for more details on the densitometric analyses). The addition of SDS had very minor effects on the intensity of protein bands in the 75- to 100-kDa range.

In Fig. 1*C*, we found that adding methanol and/or SDS to the transfer buffer increased the molecular weight range of bands. As can be seen in Fig. 1*C*, *top*, consistent with Fig. 1*B*, the addition of SDS resulted in more *O*-GlcNAc positive higher molecular weight proteins compared with no SDS. The absence of methanol decreased the intensity of midrange molecular weight *O*-GlcNAc positive proteins. In Fig. 1*C*, *bottom*, we show Coomassie-stained gels following transfer. In the absence of SDS, there is more protein remaining on the gel compared with the presence of 0.0375% SDS proteins, independent of the presence of methanol. Consequently, the addition of SDS to the transfer buffer improved the efficiency of the transfer of both high and low-molecular-weight proteins. In summary, the addition of SDS to the transfer buffer had the biggest effect on the molecular weight range of proteins detected. The differences between 0.0375 and 0.05% SDS are minor; however, in the subsequent studies, we used 10% methanol and 0.0375% SDS in the transfer buffer.

### Antibodies

**Figure 2.**
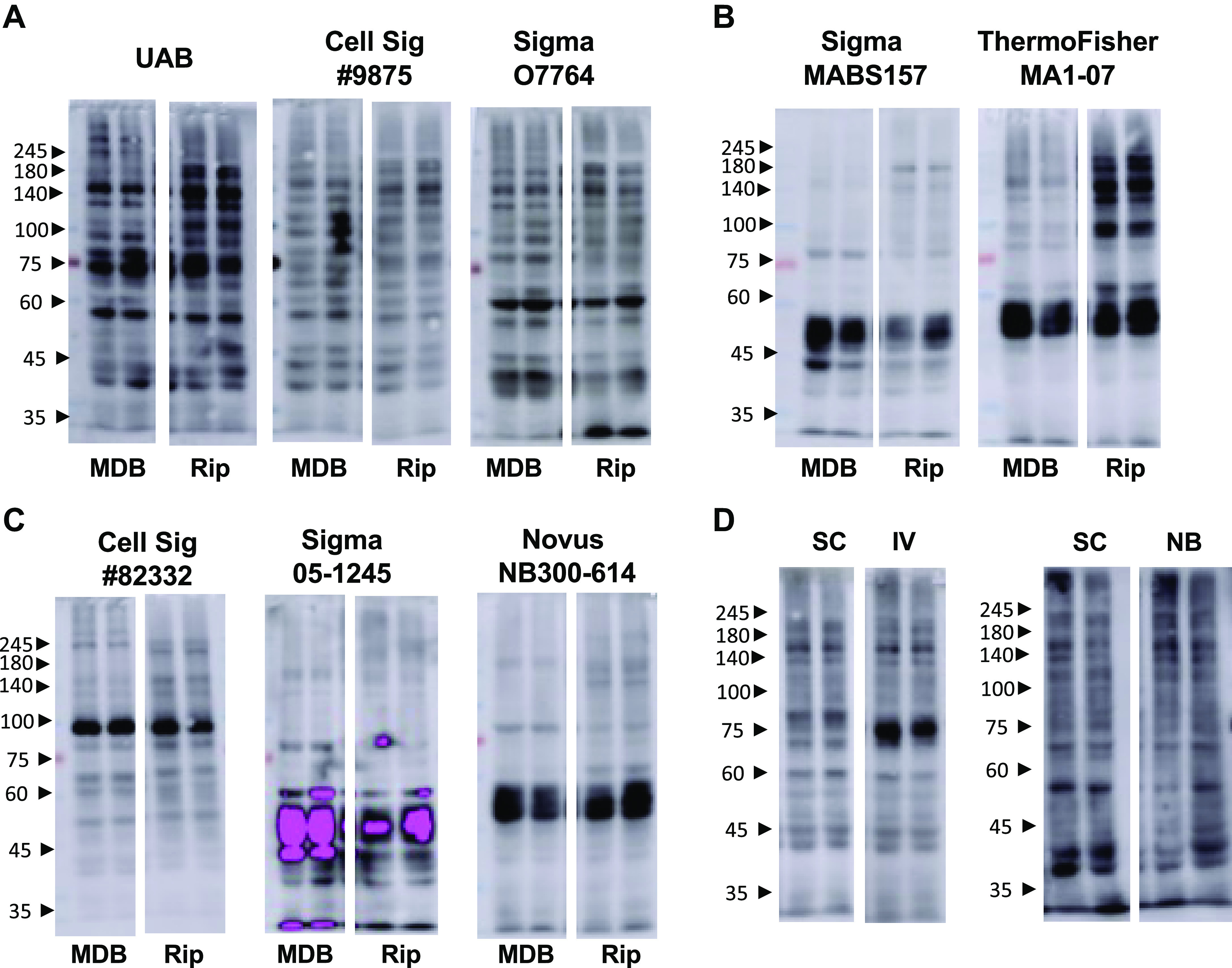
*A*: *O*-linked *N*-acetylglucosamine (*O*-GlcNAc) immunoblots of cardiac tissue extracted using either mild detergent buffer (MDB) or radioimmunoprecipitation assay (RIPA, Rip) lysis buffer, with 3% bovine serum albumin (BSA) as blocking agent and 10% methanol and 0.0375% sodium dodecyl sulfate (SDS) transfer buffer using 3 different CTD110.6 antibodies: *O*-GlcNAc immunoblots using the same protocol as in *A*, with two RL2 antibodies from different sources (*B*) and *O*-GlcNAc immunoblots using the same protocol as in *A* with 3 different *O*-GlcNAc antibodies from 3 different vendors (*C*). *D*: *O*-GlcNAc immunoblots using the same protocol as in *A* with secondary IgM antibodies from Invitrogen (IV) or Novus Biologicals (NB). Antibody details are listed in Table 1. All MDB and RIPA lysis samples were run on the same blots for each antibody. See Supplemental Fig. S3 for complete blots.

Antibody source is another potential variable that could affect the quality and variability of immunoblots; therefore, we compared our UAB-CTD110.6 with CTD110.6 from Cell Signaling and Sigma-Aldrich (Table 1), using identical lysis conditions, 3% BSA as the blocking agent, and 10% methanol plus 0.0375% SDS in the transfer buffer. As shown in Fig. 2*A*, although there are differences in intensities, all three CTD110.6 antibodies showed similar wide molecular weight banding patterns indicating that these conditions are suitable regardless of the source of CTD110.6. The differences in intensity are likely due to differences in the sensitivity of the individual antibodies. As our focus was on the molecular weight range, we did not attempt to adjust conditions to achieve identical intensity levels. One Sigma CDT110.6 clone, MABS1254, did not perform well under these conditions, resulting in a single intense band at ∼75 kDa (Supplemental Fig. S3). On closer examination, it turned out that MABS1254 was a purified mouse monoclonal IgMκ antibody. Why this k-light chain-specific IgM antibody did not work under our experimental conditions is unclear, and since other CTD110.6 antibodies worked well, we did not pursue this further. Nevertheless, the results clearly demonstrate that differences in the composition of the IgM antibody being used can have a major effect on the resulting CTD110.6 immunoblot.

Another widely used *O*-GlcNAc antibody is RL2, and using the same conditions used for CTD110.6, we found that RL2 from Sigma-Aldrich and Thermo Fisher Scientific (Table 1) did not work well under these conditions (Fig. 2*B*). As shown in Fig. 2*C*, we also tested three different *O*-GlcNAc antibodies from Cell Signaling, Sigma-Aldrich, and Novus Biologicals (Table 1); however, none of these antibodies worked as well as CDT110.6 under the same conditions.

### Nonspecific Interactions

A problem frequently encountered with CTD110.6 *O*-GlcNAc blots is an intense band at ∼75 kDa, which is a result of a nonspecific interaction of the secondary antibody with IgM (38). As can be seen in Figs. 1 and 2*A*, here the intensities of bands around 75 kDa were close to the surrounding bands. In the process of completing this study, we found out that the secondary antibody we had been using from Santa Cruz, was discontinued; consequently, it would be necessary to find an alternative secondary antibody for future studies. In Fig. 2*D*, we compared blots using the original Santa Cruz secondary with secondary antibodies from Invitrogen and Novus Biologicals (Table 1). With the use of our protocols, the Invitrogen secondary antibody exhibited a moderately intense band at ∼75 kDa, whereas the secondary antibody from Novus Biologicals resulted in blots comparable with those using Santa Cruz (Fig. 2*C*). Clearly other secondary antibodies may work equally as well as the discontinued Santa Cruz and Novus Biologicals antibodies. It is also possible that protocol modifications could result in the Invitrogen secondary antibody not exhibiting the intense ∼75 kDa band; however, we did not investigate this further.

**Figure 3.**
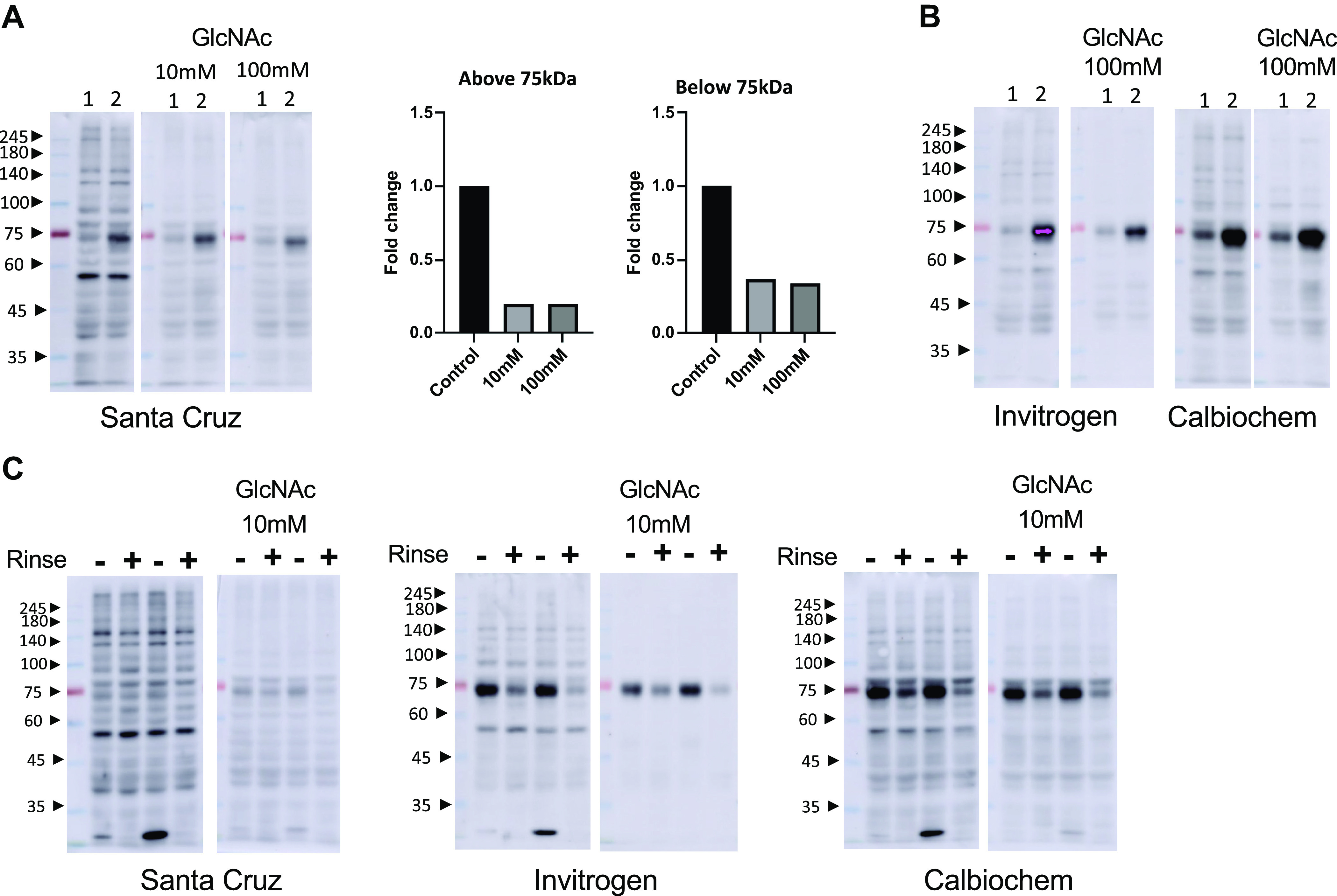
*A*, *left*: CTD110.6 *O*-linked *N*-acetylglucosamine (*O*-GlcNAc) immunoblots from extracts of two different hearts with 3% bovine serum albumin (BSA) as blocking agent and transfer buffer containing 10% methanol and 0.0375% sodium dodecyl sulfate (SDS) and Santa Cruz secondary antibody, with 0, 10, and 100 mM GlcNAc. *A*, *right*: densitometry analyses measured above and below 75 kDa. As we were comparing the effects of blocking CTD110.6 with GlcNAc, the gels were by necessity run separately. However, for image analysis purposes, the gels were merged into a single image; see text and Supplemental Fig. S6*A* for more details on the analysis. *B*: CTD110.6 *O*-GlcNAc immunoblots of cardiac extracts ± 100 mM GlcNAc with Invitrogen and Calbiochem secondary antibodies. *C*: CTD110.6 *O*-GlcNAc immunoblots from extracts of two different hearts. Each heart was divided into two, one half was immediately freeze clamped without rinsing (−) thereby maximizing blood contamination and the other was rinsed in ice cold buffer for 2–3 s (+) to reduce blood contamination before freeze clamping. The immunoblots were run under identical conditions to those in Fig. 3*A.* All samples were extracted using mild detergent buffer (MDB).

Although the bands around 75 kDa were not more intense than those immediately above or below (Fig. 2*A*), we cannot rule out the possibility that they could be due to nonspecific interactions with IgM. Therefore, we tested the specificity of the staining for *O*-GlcNAc by competing away the primary antibody with free GlcNAc as previously described (38). In Fig. 3*A*, we show the results of competing away CTD110.6 with GlcNAc using the Santa Cruz secondary antibody. Densitometric analysis indicates a ∼80% reduction in overall intensity with 10 and 100 mM GlcNAc above and below 75 kDa (see *Densitometric Analysis of O-GlcNAc Blots* and Supplemental Fig. S6*D* for more details on the densitometric analysis). A band at ∼75 kDa remained in the presence of 10 and 100 mM GlcNAc with the Santa Cruz secondary antibody that has been used throughout this study, indicating that this is a nonspecific band. In contrast, the intense band between 45 and 60 kDa is absent in the presence of GlcNAc, demonstrating its specificity. This was also the case for banding >75 kDa; surprisingly, below 60 kDa, residual staining remained, indicating that some of the intensity seen in the CTD110.6 blot at the lower molecular weight range is likely due to nonspecific interactions. Similar results were obtained using secondary antibodies from Invitrogen and Calbiochem using 100 mM GlcNAc (Fig. 3*B*).

In Fig. 3, *A* and *B*, the intensity of the ∼75-kDa band was very different between the two samples regardless of the secondary antibody, and we wondered if this could be due to minor differences during the sample processing. One factor we considered was potentially differing amounts of blood remaining in the cardiac tissue. Therefore, to determine whether blood contamination was a factor, freshly isolated hearts were divided into two halves: one was immediately freeze-clamped, and the other half was rinsed in ice-cold saline for 2–3 s before freeze-clamping. The samples were subsequently lysed as aforementioned and CTD110.6 immunoblots run with and without 10 mM GlcNAc using three different secondary antibodies. As shown in Fig. 3*C*, the intensity of the nonspecific ∼75-kDa band was higher in samples that contained more blood compared with those that were briefly rinsed, regardless of the secondary antibody being used; however, the intensity of this band was still dependent on the secondary antibody.

### Myofilament and High-Molecular-Weight Proteins

**Figure 4.**
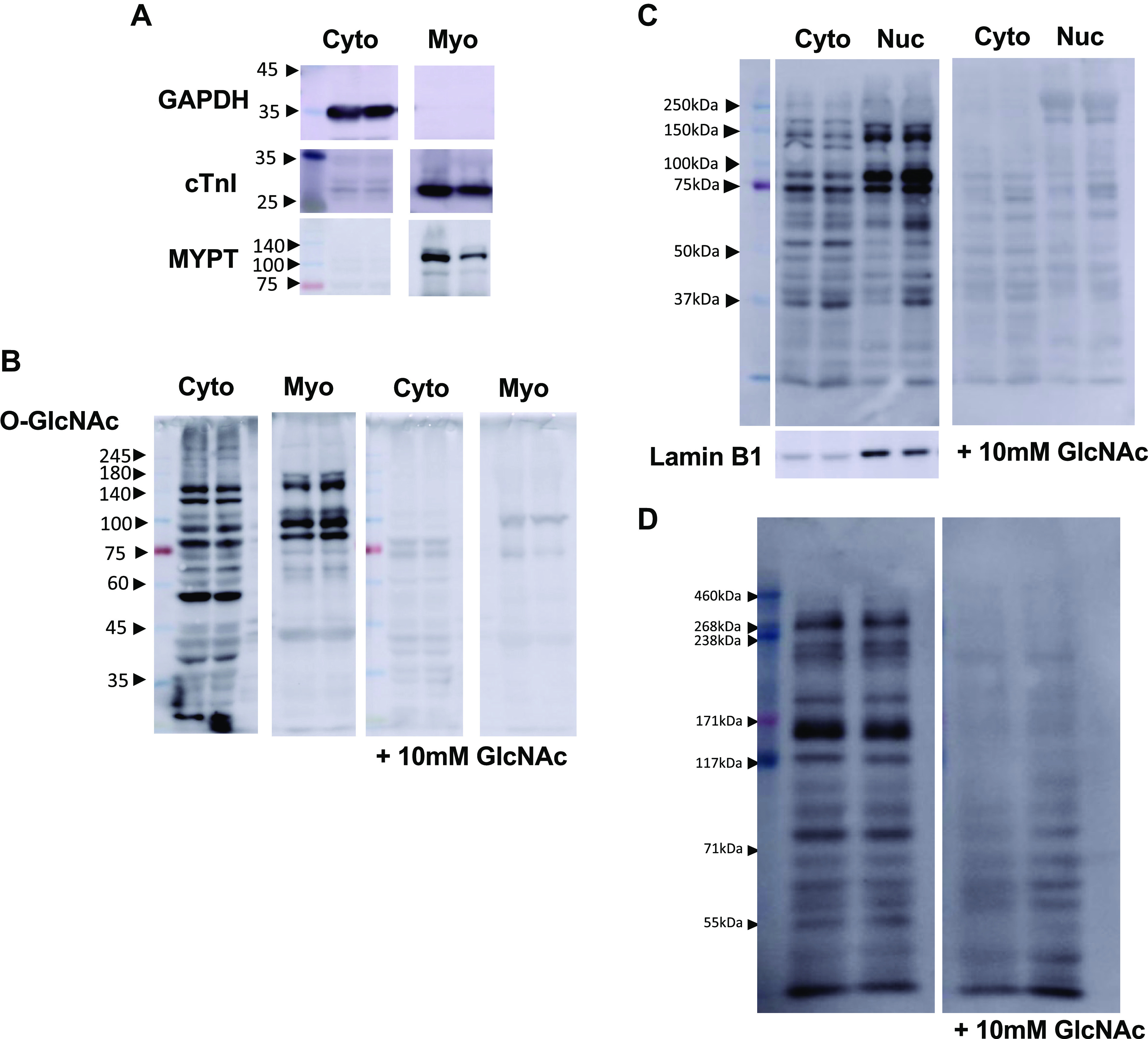
*A*: immunoblots of glyceraldhyde-3-phosphate dehydrogenase (GAPDH), cTnI, and MYTP in cytosolic (Cyto) and myofilament (Myo) fractions. *B*: *O*-linked *N*-acetylglucosamine (*O*-GlcNAc) immunoblots from the same cytosolic and myofilament fractions using CTD110.6 and competition experiment with GlcNAc demonstrating the specificity of the *O*-GlcNAcylated myofilament proteins. Note that the cytosolic and membrane fractions in *A* and *B* were run on the same blots but were cropped for ease of presentation. Uncropped blots are shown in Supplemental Fig. S4. *C*: CTD110.6 *O*-GlcNAc immunoblots of nuclear (Nuc) and cytosolic fractions, and competition experiment with GlcNAc. Lamin B1 was used as a marker for nuclear fraction. *D*: CTD110.6 *O*-GlcNAc immunoblot demonstrating high molecular weight *O*-GlcNAcylated proteins up to ∼450 kDa. Note that GlcNAc competition experiments in *C* and *D* were by necessity run on separate gels from the primary CTD110.6 gels.

Myofilament proteins in the heart have been shown to be *O*-GlcNAcylated by mass spectrometry (40, 41), and CTD110.6 immunohistochemistry of the rat exhibited a clear striated pattern, consistent with *O*-GlcNAcylation of myofilament proteins (24). However, the extent to which cardiac myofilament proteins can be detected via CTD110.6 immunoblot has not been evaluated, which is relevant given the large amount of myofilament proteins in the heart. Therefore, we prepared cytosolic and myofilament enriched fractions, as aforementioned, using the MDB and RIPA lysis buffers, as well as the lysis buffer used by Yin et al. (37). In Fig. 4*A*, we show enrichment of both cTnI and myosin phosphatase-targeting subunit (MYTP) in the myofilament fraction with minimal contamination from the cytosolic fraction in samples processed with the MDB lysis buffer. In Fig. 4*B*, we show CTD110.6 positive bands in the myofilament fraction between 180 and 74 kDa, which are absent in the presence of 10 mM GlcNAc. Thus, a limited subset of *O*-GlcNAcylated myofilament proteins is detected by CTD110.6; however, other pan-*O*-GlcNAc antibodies may be able to detect a broader range of myofilament proteins. The fractionation protocol used should yield cytosolic, membrane, and myofilament fractions (37); therefore, although our focus was on the myofilament fraction, we also looked at the membrane fraction. As shown in Supplemental Fig. S4, the membrane fraction was only cleanly isolated using the lysis buffer of Yin et al. (37). Several CTD110.6 positive bands were observed in the membrane fraction, which were absent in the presence of UDP-GlcNAc, indicating the presence of *O*-GlcNAcylated proteins in this fraction. As RIPA lysis buffer is commonly used, we carried out the same fractionation protocol following tissue lysis with RIPA. However, as can be seen in Supplemental Fig. S4, this tissue fractionation protocol did not work when RIPA lysis buffer was used. We also tested a different nuclear fractionation protocol, as aforementioned, and demonstrated a similar wide range of molecular weight *O*-GlcNAc proteins detected in both fractions that were absent in the presence of GlcNAc (Fig. 4*C*).

In most studies, the upper molecular weight range reported for *O*-GlcNAc blots is ∼250 kDa; however, there are many proteins at a higher molecular weight that are usually not examined. In an unrelated study, we have been optimizing conditions for immunoblots of higher molecular weight proteins such as dystrophin (∼400 kDa). Therefore, following that approach, we used RIPA lysis buffer, combined with a 6% SDS gel followed by transfer including 0.0375% SDS-10% methanol. We were able to identify CTD110.6 positive proteins up to ∼450 kDa that were absent in the presence of 10 mM GlcNAc (Fig. 4*D*), indicating that it is possible to detect higher molecular weight *O*-GlcNAcylated proteins. To successfully immunoblot proteins >500 kDa, urea-based extraction buffers and SDS-agarose gels are typically used (42). As these are very different from the conditions used here, we did not look at *O*-GlcNAcylation of very-high molecular weight proteins.

### Storage Duration

**Figure 5.**
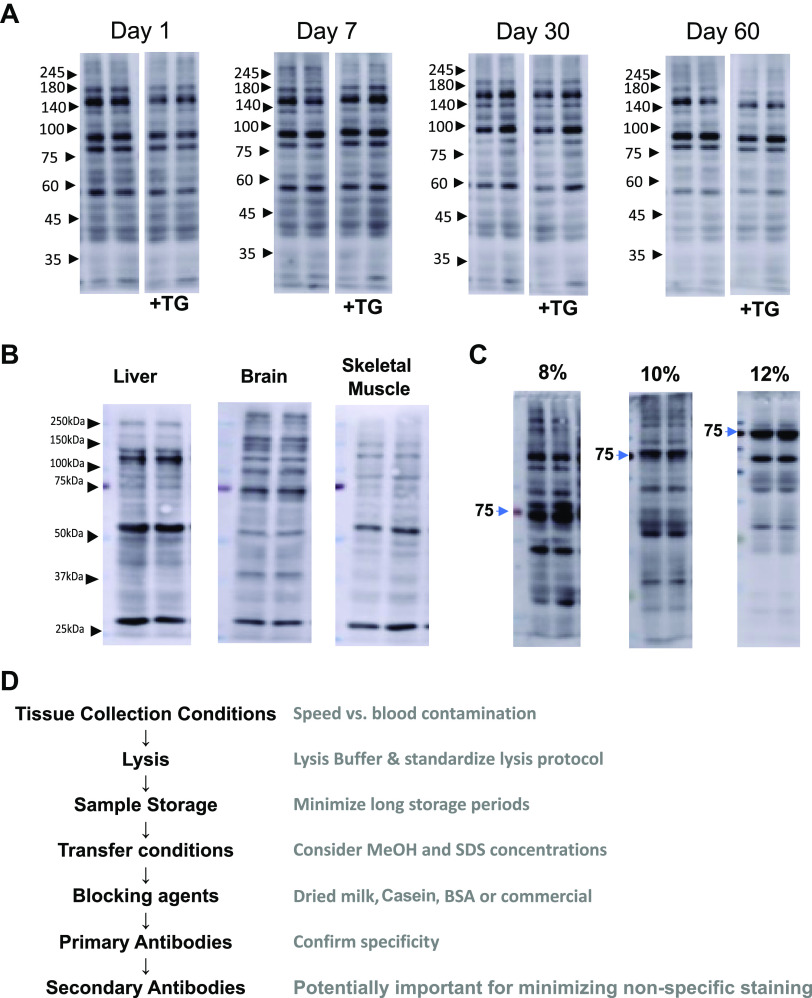
*A*: *O*-linked *N*-acetylglucosamine (*O*-GlcNAc) immunoblots of cardiac tissue extracted using radioimmunoprecipitation assay (RIPA) lysis buffer, with 3% bovine serum albumin (BSA) as blocking agent and transfer buffer containing 10% methanol and 0.0375% sodium dodecyl sulfate (SDS), stored at −80°C for 1, 7, 30, and 60 days with and without 10 µM of Thiamet-G (TG). These were separate aliquots of the same sample stored for 1–60 days to avoid potential adverse effects of freeze-thawing on the sample. The same results were observed with mild detergent buffer (MDB) lysis, see Supplemental Fig. S5. *B*: *O*-GlcNAc immunoblots of skeletal muscle (gastrocnemius), liver, and brain using MDB, with 3% BSA as blocking agent and transfer buffer containing 10% methanol and 0.0375% SDS using an 8% gel. *C*: *O*-GlcNAc immunoblots using the same protocol as in *A* using 8, 10, and 12% gels. *D*: schematic summarizing the key elements of protocol optimization for pan-*O*-GlcNAc immunoblots to include a wide range of molecular weights of detected proteins and minimization of the nonspecific 75-kDa band.

It is typically assumed that storing samples at −80°C will keep the proteins in a stable state for several months; however, whether this is the case for post-translational modifications is less clear. For example, Utter et al. reported that phosphorylation of specific myofilament proteins was significantly reduced after only 30 days of storage and some after as little as 7 days of storage at −80°C (43). Therefore, following the protocol described by Utter et al., (43) lysates were diluted into sample buffer before storing at −80°C, and *O*-GlcNAc levels in the same samples were determined at 1, 7, 30, and 60 days of storage in the presence and absence of the OGA inhibitor Thiamet-G (Fig. 5*A*). There are no clear changes in *O*-GlcNAc levels or banding patterns between 1 and 7 days (Fig. 5*A*); however, by 30 days, there were some noticeable changes. For example, the intense band just above the 75-kDa molecular weight marker at 1 and 7 days is much less intense after 30 days of storage. The regions immediately between 65- and 45-kDa markers are also less intense after 30 days. By 60 days of storage, there were further decreases in intensity in different regions. The addition of Thiamet-G did not prevent the loss in *O*-GlcNAc levels at any time point, which is not surprising as samples were stored in a sample buffer.

### Other Tissues

Although our focus has been on optimizing *O*-GlcNAc blots from whole heart tissue, we wanted to determine whether the same protocol was effective in other tissues. In Fig. 5*B*, we show CTD110.6 immunoblots from skeletal muscle, liver, and brain using regular lysis buffer. In all cases, using 3% BSA as the blocking agent combined with 10% methanol-0.0375% SDS transfer buffer resulted in the detection of *O*-GlcNAc positive bands spanning the range from ∼25 to ∼250 kDa regardless of the lysis buffer. However, the overall intensity is not as uniform as that seen with the heart *O*-GlcNAc blots, suggesting additional optimization steps maybe required for other tissues.

### Choice of %Gel

When examining changes in individual proteins, the %gel used is typically tailored to the molecular weight of that protein. In evaluating changes in overall *O*-GlcNAc levels across a broad molecular weight range, the choice of %gel is less clear. Therefore, we compared different gels ranging from 8 to 12%, and the results suggest that 8 and 10% gels provide a broader range of CTD110.6 positive bands compared with 12% gels (Fig. 5*C*) unless studies are focused on higher molecular weight proteins (Fig. 4*D*).

### Densitometric Analysis of O-GlcNAc Blots

In the analysis of single protein immunoblots, the intensity of the protein band can frequently be obtained without needing to consider contributions of background intensity from the gel. However, in most cases, the densitometric analysis of *O*-GlcNAc blots is usually performed on the whole lane or over a specific range of molecular weights. In such an analysis, the background intensity can have a marked effect on the overall quantification and can thus potentially influence the interpretation of the results. We routinely use the widely available ImageJ analysis software (44) for our densitometric analysis of immunoblots; however, without any background reduction analysis of the 140–180-kDa region indicated minimal changes in intensity with SDS (Supplemental Fig. S6*A*), even though visually there is clearly a marked increase, demonstrating the importance of background reduction. We then tried the “rolling ball” background subtraction feature in ImageJ, which is designed to correct for uneven background intensity. However, consistent with an earlier report (45), we found that the rolling ball algorithm resulted in results that were worse than no background correction. Therefore, we decided to take an approach where the intensity was subtracted that covered the “lowest” signals among all the lanes, which we found to be the more reliable approach for our data. The placement of the line to indicate the signal to be subtracted is somewhat arbitrary, and one can take a more conservative or aggressive approach as shown in Supplemental Fig. S6, *A–C*. In the data shown in Fig. 1*B*, we used the more aggressive approach. We did not use any background reduction in the data shown in Fig. 3*B* because the difference in intensity of the blots with GlcNAc was low and already very close to baseline (Supplemental Fig. S6*D*). Other image software packages such as Image Studio Lite or Empiria Studio (LI-COR Biosciences) and Image Lab (Bio-Rad) are also widely used but were not tested here because of compatibility problems with third-party images. Regardless of the software used for image analysis, it is important to consider the methods for the reduction of background in the densitometry of *O*-GlcNAc immunoblots and understand the assumptions underlying these methods. The decision to use background reduction may also depend on the intensities of the blots being analyzed relative to the background intensity.

A list of all the different procedures tested at each step during the optimization process and their outcomes is shown in Table 3.

**Table 3.** List of all the different components tested at each step during the optimization process highlighting key elements to be considered for future studies

Process	Elements Tested	Observations	Recommendations
Tissue processing	Rinse to reduce blood contamination	Rinsing heart tissue in ice-cold saline for 2–3 s before freeze clamping reduced nonspecific 75-kDa band compared with no removal of blood.	Reducing blood contamination by rinsing tissue in ice-cold saline may help reduce the intensity of the nonspecific 75-kDa band. This may be particularly relevant for the heart due to the relatively large amount of blood in the ventricles, as well as the vasculature.
Lysis buffers	MDB, RIPA	Had no effect on the molecular weight range of proteins detected.	The lysis buffers tested do not appear to affect the quality of *O*-GlcNAc blots. If other types of lysis buffers, i.e., urea-based are used these would need further testing.
Blocking agents	Casein, BSA	Both worked well; BSA resulted in slightly sharper bands. Neither affected the molecular weight range of proteins detected.	Other blocking agents may work as well or better but were not tested. Testing different blocking agents strongly suggested if the quality of *O*-GlcNAc blots needs improving, such as reducing background intensity and nonspecific antibody interactions.
Transfer buffers	Methanol, SDS	The addition of 10% methanol to the transfer buffer resulted in modest improvements. The addition of ∼0.03–0.05% SDS markedly improved the transfer of high molecular weight proteins.	If *O*-GlcNAc-positive proteins are not being detected between 100 and 250 kDa, the addition of a low percent SDS to the transfer buffer should be considered.
			
Primary Antibodies	CTD110.6, RL2, others (see Table 1)	This protocol was optimized for CTD110.6 and did not work for other pan-*O*-GlcNAc antibodies. Of note, a purified IgMk-CTD110.6 antibody also did not work using this protocol.	If using CTD110.6 antibody and the expected molecular weight range is not observed, or nonspecific band(s) dominate, consider trying a new source of antibody.
			Other *O*-GlcNAc antibodies may work just as well, but different conditions will likely be needed to achieve similar-quality blots.
Secondary Antibodies	Numerous (see Table 1)	The relative intensity of the nonspecific band at 75 kDa varied, and this depended, at least in part, on the source of the secondary antibody. The reason for this was not determined.	If CTD110.6 blot is dominated by the intense band at 75 kDa, different secondary antibodies should be tested. The use of a preclearing step, with Protein A/G agarose beads might also be helpful in preventing this nonspecific band, but this was not tested here.
			
Specificity	GlcNAc	10–100 mM GlcNAc was sufficient to prevent most CDT110.6 positive bands from ∼30 to 450 kDa, demonstrating specificity. Some residual staining at lower molecular weights was observed, and the nonspecific 75-kDa band was not blocked.	Even when banding intensity in the 75-kDa region is close to surrounding bands, it is important to perform a GlcNAc competition assay to determine the extent to which nonspecific interactions remain.
			
Sample storage	Duration of storage at −80°C from 1–60 days	Over a 60-day period, lysates diluted and stored in sample buffer at −80°C exhibited gradual changes in intensity of *O*-GlcNAc positive bands.	Recommendations: *1*) Carry out *O*-GlcNAc immunoblots as soon as feasible following lysis. *2*) Store lysates in the sample buffer. *3*) Track the storage time of samples. *4*) Avoid comparisons of samples stored for different time periods.
Densitometric analyses	Background reduction	ImageJ was used for the densitometric analyses, and several approaches were used to reduce background intensity. As previously reported (45), we found that the ImageJ rolling ball algorithm led to misleading results in quantifying CTD110.6 *O*-GlcNAc blots.	Numerous factors can contribute to high and variable background intensities. Although some of these are due to technical reasons, background reduction approaches may be necessary for densitometric analyses of whole or partial lanes of *O*-GlcNAc blots.
			There is no specific guidance as to the best approach for background reduction; therefore, explicit reporting of the approach used is strongly encouraged.

MDB, mild detergent buffer; RIPA, radioimmunoprecipitation assay; *O*-GlcNAc, *O*-linked *N*-acetylglucosamine; BSA, bovine serum albumin; SDS, sodium dodecyl sulfate.

## DISCUSSION

The first pan *O*-GlcNAc antibody, RL2 was described in 1987 (46), this was followed by HGAC-85 in 1990 (47), and CTD110.6 in 2001 (48). CTD110.6 was shown to identify *O*-GlcNAc modifications specifically on serine and threonine residues in contrast to HGAC-85 which identified other carbohydrates, and CTD110.6 typically identified a wider range of proteins than RL2 (48). Other *O*-GlcNAc antibodies are also commercially available, such as the anti-*O*-GlcNAc clone 9D1.E4 (49) from Sigma-Aldrich (Cat. No. 82332) and the *O*-GlcNAc MultiMab from Cell Signaling (Cat. No. 05–1245); however, currently, these are not as widely used as CTD110.6 or RL2. Chemoenzymatic labeling techniques have also been developed in recent years, although additional sample processing steps are typically needed. Consequently, CTD110.6 and RL2 remain widely used in the analysis of changes in overall *O*-GlcNAc levels in numerous cells and tissues including the heart.

There has been growing interest in evaluating global *O*-GlcNAc levels in the heart because of its potential role in mediating various pathological conditions including ischemia-reperfusion injury (23, 24, 26), diabetic cardiomyopathy (9, 10, 13, 36, 50, 51), cardiac hypertrophy (19, 20, 52, 53), arrhythmias (54, 55), and heart failure (14, 18–20, 56, 57). However, despite their widespread use, potential limitations that occur with both CTD110.6 and RL2 include the detection of a limited number of *O*-GlcNAc positive bands, the considerable variability in the number and molecular weight range of proteins detected, and the frequent presence of an intense nonspecific IgM band at ∼75 kDa (CTD110.6) and IgG band at ∼55 kDa (RL2). In many of our earlier studies, we did not observe *O*-GlcNAc positive bands below ∼50 kDa and we have had inconsistent detection of proteins >110 kDa (11, 24, 35). The lack of detection of proteins over specific molecular weight ranges was frequently attributed to “epitope specificity” of the antibody (58) and/or low abundance of *O*-GlcNAc-modified proteins, particularly in the lower molecular weight range. Although these are potential explanations, they do not account for the considerable variability of banding patterns reported in published cardiac *O*-GlcNAc immunoblots within the same laboratory or between laboratories using the same antibodies. Therefore, our goal was to optimize a protocol that enhanced the molecular weight range of detection thereby increasing the quality and standardizing the reproducibility of cardiac *O*-GlcNAc immunoblots. This study is meant as a guideline to some of the key technical variables we identified, to produce more reliable results in the identification of *O*-GlcNAc positive proteins spanning as broad a molecular weight range as possible, using the CTD110.6 antibody.

We focused on CTD110.6 as it remains the most widely used pan-*O*-GlcNAc antibody to evaluate changes in overall *O*-GlcNAc levels in the heart and other tissues. In addition, CTD110.6 has also been the mainstay of our own work for many years; nevertheless, we have observed inconsistencies in our own results. We considered two different types of lysis buffers, two different blocking agents, and the composition of the transfer buffer. We found that there was no difference regarding the molecular weight range of detected *O*-GlcNAcylated proteins, between a mild detergent buffer (MDB) and the stringent denaturing RIPA buffer (Fig. 1), although the banding patterns are different due to different protein populations sampled by the different lysis buffers. We concluded that BSA was a slightly better-blocking agent than casein, although the differences were subtle. Commercially available blocking buffers may be just as effective as or better than BSA; however, we chose not to test them because their composition is proprietary.

We found that the addition of 0.03–0.05% SDS to the transfer buffer resulted in a more efficient transfer of proteins at both the high and low molecular weight range resulting in reproducible cardiac *O*-GlcNAc blots with protein banding patterns ranging from ∼30 to ∼250 kDa. It should be noted, however, that some of the *O*-GlcNAc positive staining below ∼50 kDa appears to be nonspecific as it was still visible in the GlcNAc competition experiments (Fig. 3). We found that this protocol worked well for CTD110.6 antibodies from different sources, but it did not work as well with RL2 or several other commercially available *O*-GlcNAc antibodies. Of note, a κ-light chain-specific IgM CTD110.6 antibody did not work as well as other IgM antibodies (Supplemental Fig. S3*A*). Although the reason for this remains unclear, there are significant physiochemical differences between κ- and λ-light chains (59), which could account for these observations. Therefore, protocols will be needed to be optimized for different *O*-GlcNAc antibodies as well as adjusted, depending on the tissue source to achieve *O*-GlcNAc blots covering a similar wide range of molecular weights as those observed here in cardiac tissue. In addition to different *O*-GlcNAc antibodies, there are also Click-iT labeling and Biotin detection approaches, which may have advantages over antibodies as they are epitope agnostic. However, since our goal here was to optimize and troubleshoot conditions for a widely used pan-*O*-GlcNAc antibody rather than compare different techniques for detecting *O*-GlcNAcylated proteins, we did not examine nonantibody approaches.

A frequent limitation with CTD110.6 blots is the presence of an intense band at ∼75 kDa, due to nonspecific interaction of the secondary antibody with IgM (38). This nonspecific band can be particularly problematic when trying to quantify overall *O*-GlcNAc levels because depending on its intensity it can adversely affect the visualization of lower-intensity bands and thus potentially obscure changes between groups. We found that the intensity of the nonspecific band was dependent in part on the secondary antibody being used (Figs. 2*C* and 3). However, even when the intensity of the band at 75 kDa was close to the surrounding bands, it was not blocked by competition with GlcNAc, demonstrating that nonspecific binding remains (Fig. 3*A*); although, reducing blood contamination of the cardiac tissue sample appeared to reduce the intensity of this band (Fig. 3*C*). It is possible, therefore, that differences in the blood content of cardiac tissue could be a factor contributing to the variability in published *O*-GlcNAc blots. The GlcNAc competition experiments demonstrated that proteins >75 kDa were clearly *O*-GlcNAcylated proteins, while below 60 kDa there was some residual staining in the presence of up to 100 mM GlcNAc. This is highlighted by the fact that the intense band just below 60 kDa was completely blocked by GlcNAc. Consequently, analysis and interpretation of lower molecular weight CTD110.6 positive bands need to be conducted with caution. Preclearing, using protein A and/or G agarose beads, may be a useful approach to help reduce nonspecific banding because of blood contamination or the effects of secondary antibodies. Other reagents such as anti-mouse IgM Dynabeads (Thermo Fisher Scientific Cat. No. 11039 D) could also be valuable tools for decreasing nonspecific interactions with CTD110.6. Because of the potential variability of banding patterns, and the intensity of the nonspecific bands, we believe that it is essential that a molecular weight ladder be included with all pan-*O*-GlcNAc blots.

We also showed that this protocol was able to identify some *O*-GlcNAcylated myofilament proteins between 75 to 180 kDa (Fig. 4, *A* and *B*); however, other fractionation protocols or *O*-GlcNAc antibodies may detect a wider range of myofilament proteins. It should be noted that only mild detergent-based buffers were effective in isolating the myofilament proteins as the RIPA lysis buffer did not work with the fractionation protocol used here (Supplemental Fig. S4). In addition, a wide molecular weight range of proteins was detected in both cytosolic and nuclear fractions, and we were able to detect *O*-GlcNAcylated proteins up to ∼450 kDa (Fig. 4, *C* and *D*). This demonstrates that this protocol works effectively for a range of different types of proteins. The ability to detect *O*-GlcNAcylated proteins up to ∼450 kDa indicates the possibility of detecting important metabolic proteins such as mTOR (∼300 kDa) as well as cytoskeletal proteins such as dystrophin (∼450 kDa). Further protocol development will be needed to detect *O*-GlcNAcylation of very high molecular weight proteins such as the dual kinase obscurin (∼870 kDa) and titin (>3,500 kDa). Such efforts would be worthwhile given the importance of such proteins in the heart.

In studies of phosphorylation of specific myofilament proteins, significant reductions in some phosphorylation sites were observed in samples stored for as little as 7 days at −80°C (43). We show here that lysates stored in sample buffer at −80°C showed no obvious changes between 1 and 7 days of storage although there were some minor changes by 30 days, which became more pronounced by 60 days. The addition of the OGA inhibitor Thiamet-G did not prevent the loss of *O*-GlcNAc levels over this 60-day period. Therefore, to ensure optimal reproducibility *O*-GlcNAc blots, we recommend that they should be run as soon as feasible following tissue lysis and that the duration of storage be noted to ensure that samples stored for similar periods of time can be compared. More generally our results and those of Utter et al. demonstrate that protein PTMs may be less stable at −80°C than typically assumed. It is worth noting that Utter et al. reported that the type of anesthesia used also altered myofilament phosphorylation (43). It remains to be determined whether this also affects cardiac *O*-GlcNAc levels.

We have not attempted to evaluate all possible variables that could affect overall *O*-GlcNAc immunoblot quality, such as different types of membranes, commercial blocking buffers, or different transfer methods such as the rapid semidry transfer methods (e.g., Bio-Rad Trans-Blot Turbo Transfer System and Invitrogen Power Blotter), type and duration of anesthesia before tissue isolation. Rather we have focused on relatively minor adjustments to already established protocols in our laboratories. Additional, important technical details regarding CTD110.6 immunoblots are described elsewhere (38). In Fig. 5*D*, we summarize the key elements that we identified as being important to consider when optimizing an *O*-GlcNAc immunoblot. Table 3 contains a detailed summary of all the alternatives tested for each step in the protocol optimization process in addition to recommendations of factors to be considered when optimizing a new pan-*O*-GlcNAc immunoblot protocol.

While we have demonstrated that it is possible, using CTD110.6, to generate reproducible pan-*O*-GlcNAc immunoblots of murine cardiac tissue extracts with a banding pattern that covers a molecular weight range of ∼30–450 kDa. There is no reason that this should not be possible with other commercially available *O*-GlcNAc antibodies. Our findings also illustrate the importance of including detailed methodological information for immunoblots beyond just identifying the source of the antibody being used, including but not limited to blocking agents, transfer buffers, and specific transfer protocols (Fig. 5*D*). To appropriately quantify cardiac CDT110.6 blots, the GlcNAc competition protocol should be used to determine the contribution of the nonspecific IgM band and if necessary to exclude this region during densitometric analysis. We also note that densitometric analysis of *O*-GlcNAc blots often requires the use of some form of background reduction algorithm. It is necessary to understand the underlying assumptions in such algorithms and the specific approach used should be reported.

Thus, in conclusion, we have shown that it is possible to obtain pan-*O*-GlcNAc blots of cardiac tissue using the widely available CTD110.6 antibody, which minimizes commonly recognized limitations such as limited molecular weight range coverage and the intensity of the nonspecific IgM band at ∼75 kDa. We also highlight the importance of processing cardiac lysates in a prompt manner, with minimal delays in diluting the lysates in sample loading buffer and the use of GlcNAc competition studies to ensure that only *O*-GlcNAc specific protein bands are quantified. Although our focus in this study was on CTD110.6, other commercially available antibodies may perform as well if not better, following protocol optimization tailored to a specific antibody. We have demonstrated that reproducible cardiac *O*-GlcNAc immunoblots, spanning a molecular weight range from ∼30–450 kDa, are possible with relatively minor protocol adjustments and that application of the principles described here should help improve the reproducibility of *O*-GlcNAc immunoblots as well as facilitate comparisons across studies. Although our studies here focused on the heart, there is no reason to believe that these same principles should not apply to the study of *O*-GlcNAcylation in other tissues with additional protocol modifications.

## DATA AVAILABILITY

Data will be made available upon reasonable request.

## SUPPLEMENTAL DATA

10.6084/m9.figshare.23589084 Supplemental Figs.S1–S6: https://doi.org/10.6084/m9.figshare.23589084.

## GRANTS

This work was supported in part by National Heart, Lung, and Blood Institute Grant Nos. R21HL152354 (to J.C.C. and A.R.W.), R01HL133011 (to A.R.W.), and R01HL149354 (to J.C.C.) and American Heart Association Postdoctoral Fellowship 834132 (to C.H.).

## DISCLOSURES

No conflicts of interest, financial or otherwise, are declared by the authors.

## AUTHOR CONTRIBUTIONS

A.R.W. and J.C.C. conceived and designed research; L.Z., D.Z., and C-M.H. performed experiments; L.Z. analyzed data; L.Z., A.R.W., and J.C.C. interpreted results of experiments; L.Z., C-M.H., and J.C.C. prepared figures; J.C.C. drafted manuscript; L.Z., D.Z., C-M.H., A.R.W., and J.C.C. edited and revised manuscript; L.Z., D.Z., A.R.W., and J.C.C. approved final version of manuscript.

## ENDNOTE

Note added in proof: **Narayanan B, Zahra F, Reeves RA, Aggarwal A, O'Meally RN, Henry RK, Craven M, Jacobson A, Cole RN, Kohr MJ, Umapathi P, Zachara NE.** Differential Detection of O-GlcNAcylated proteins in the heart using antibodies. *Anal Biochem* 678: 115262, 2023. doi:10.1016/j.ab.2023.115262.

Narayanan et al. reported that the RL2 antibody exhibits greater reactivity to contractile proteins than CTD110.6. Here we show that CTD110.6 recognizes myofibrillar proteins between ∼100–180 kDa (Fig. 4*B*); however, we did not compare differences in reactivity between CTD110.6 and RL2.

## References

[1] Torres CR, Hart GW. Topography and polypeptide distribution of terminal *N*-acetylglucosamine residues on the surfaces of intact lymphocytes. Evidence for O-linked GlcNAc. J Biol Chem 259: 3308–3317, 1984. 6421821

[2] Chatham JC, Zhang J, Wende AR. Role of O-linked N-acetylglucosamine protein modification in cellular (patho)physiology. Physiol Rev 101: 427–493, 2021. doi:10.1152/physrev.00043.2019. 32730113 PMC8428922

[3] Hart GW, Slawson C, Ramirez-Correa G, Lagerlof O. Cross talk between O-GlcNAcylation and phosphorylation: roles in signaling, transcription, and chronic disease. Annu Rev Biochem 80: 825–858, 2011. doi:10.1146/annurev-biochem-060608-102511. 21391816 PMC3294376

[4] Akimoto Y, Kreppel LK, Hirano H, Hart GW. Hyperglycemia and the O-GlcNAc transferase in rat aortic smooth muscle cells: elevated expression and altered patterns of O-GlcNAcylation. Arch Biochem Biophys 389: 166–175, 2001. doi:10.1006/abbi.2001.2331. 11339805

[5] Du XL, Edelstein D, Dimmeler S, Ju Q, Sui C, Brownlee M. Hyperglycemia inhibits endothelial nitric oxide synthase activity by posttranslational modification at the Akt site. J Clin Invest 108: 1341–1348, 2001. doi:10.1172/JCI11235. 11696579 PMC209429

[6] Du XL, Edelstein D, Rossetti L, Fantus IG, Goldberg H, Ziyadeh F, Wu J, Brownlee M. Hyperglycemia-induced mitochondrial superoxide overproduction activates the hexosamine pathway and induces plasminogen activator inhibitor-1 expression by increasing Sp1 glycosylation. Proc Natl Acad Sci USA 97: 12222–12226, 2000. doi:10.1073/pnas.97.22.12222. 11050244 PMC17322

[7] Liu K, Paterson AJ, Chin E, Kudlow JE. Glucose stimulates protein modification by O-linked GlcNAc in pancreatic beta cells: linkage of O-linked GlcNAc to beta cell death. Proc Natl Acad Sci USA 97: 2820–2825, 2000. doi:10.1073/pnas.97.6.2820. 10717000 PMC16013

[8] Wells L, Vosseller K, Hart GW. A role for N-acetylglucosamine as a nutrient sensor and mediator of insulin resistance. Cell Mol Life Sci 60: 222–228, 2003. doi:10.1007/s000180300017. 12678487 PMC11138838

[9] Banerjee PS, Ma J, Hart GW. Diabetes-associated dysregulation of O-GlcNAcylation in rat cardiac mitochondria. Proc Natl Acad Sci USA 112: 6050–6055, 2015. doi:10.1073/pnas.1424017112. 25918408 PMC4434690

[10] Clark RJ, McDonough PM, Swanson E, Trost SU, Suzuki M, Fukuda M, Dillmann WH. Diabetes and the accompanying hyperglycemia impairs cardiomyocyte cycling through increased nuclear O-GlcNAcylation. J Biol Chem 278: 44230–44237, 2003. doi:10.1074/jbc.M303810200. 12941958

[11] Fülöp N, Mason MM, Dutta K, Wang P, Davidoff AJ, Marchase RB, Chatham JC. Impact of Type 2 diabetes and aging on cardiomyocyte function and O-linked N-acetylglucosamine levels in the heart. Am J Physiol Cell Physiol 292: C1370–C1378, 2007. doi:10.1152/ajpcell.00422.2006. 17135297

[12] Makino A, Dai A, Han Y, Youssef KD, Wang W, Donthamsetty R, Scott BT, Wang H, Dillmann WH. O-GlcNAcase overexpression reverses coronary endothelial cell dysfunction in type 1 diabetic mice. Am J Physiol Cell Physiol 309: C593–C599, 2015. doi:10.1152/ajpcell.00069.2015. 26269457 PMC4628934

[13] Wende AR, Schell JC, Ha CM, Pepin ME, Khalimonchuk O, Schwertz H, Pereira RO, Brahma MK, Tuinei J, Contreras-Ferrat A, Wang L, Andrizzi CA, Olsen CD, Bradley WE, Dell’Italia LJ, Dillmann WH, Litwin SE, Abel ED. Maintaining myocardial glucose utilization in diabetic cardiomyopathy accelerates mitochondrial dysfunction. Diabetes 69: 2094–2111, 2020. doi:10.2337/db19-1057. 32366681 PMC7506832

[14] Lunde IG, Aronsen JM, Kvaløy H, Qvigstad E, Sjaastad I, Tønnessen T, Christensen G, Grønning-Wang LM, Carlson CR. Cardiac O-GlcNAc signaling is increased in hypertrophy and heart failure. Physiol Genomics 44: 162–172, 2012. doi:10.1152/physiolgenomics.00016.2011. 22128088

[15] Mailleux F, Gélinas R, Beauloye C, Horman S, Bertrand L. O-GlcNAcylation, enemy or ally during cardiac hypertrophy development? Biochim Biophys Acta 1862: 2232–2243, 2016. doi:10.1016/j.bbadis.2016.08.012. 27544701

[16] Zhu WZ, Ledee D, Olson AK. Temporal regulation of protein O-GlcNAc levels during pressure-overload cardiac hypertrophy. Physiol Rep 9: e14965, 2021. doi:10.14814/phy2.14965. 34337900 PMC8326887

[17] Zhu WZ, Palazzo T, Zhou M, Ledee D, Olson HM, Pasa-Tolic L, Olson AK. First comprehensive identification of cardiac proteins with putative increased O-GlcNAc levels during pressure overload hypertrophy. PLoS One 17: e0276285, 2022. doi:10.1371/journal.pone.0276285. 36288343 PMC9605332

[18] Muthusamy S, DeMartino AM, Watson LJ, Brittian KR, Zafir A, Dassanayaka S, Hong KU, Jones SP. MicroRNA-539 is up-regulated in failing heart, and suppresses O-GlcNAcase expression. J Biol Chem 289: 29665–29676, 2014. doi:10.1074/jbc.M114.578682. 25183011 PMC4207981

[19] Tran DH, May HI, Li Q, Luo X, Huang J, Zhang G, Niewold E, Wang X, Gillette TG, Deng Y, Wang ZV. Chronic activation of hexosamine biosynthesis in the heart triggers pathological cardiac remodeling. Nat Commun 11: 1771, 2020. doi:10.1038/s41467-020-15640-y. 32286306 PMC7156663

[20] Umapathi P, Mesubi OO, Banerjee PS, Abrol N, Wang Q, Luczak ED, Wu Y, Granger JM, Wei AC, Reyes Gaido OE, Florea L, Talbot CC Jr, Hart GW, Zachara NE, Anderson ME. Excessive O-GlcNAcylation causes heart failure and sudden death. Circulation 143: 1687–1703, 2021 [Erratum in *Circulation* 143: e892, 2021]. doi:10.1161/CIRCULATIONAHA.120.051911. 33593071 PMC8085112

[21] Banerjee PS, Lagerlöf O, Hart GW. Roles of O-GlcNAc in chronic diseases of aging. Mol Aspects Med 51: 1–15, 2016. doi:10.1016/j.mam.2016.05.005. 27259471

[22] Fülöp N, Feng W, Xing D, He K, Nőt LG, Brocks CA, Marchase RB, Miller AP, Chatham JC. Aging leads to increased levels of protein O-linked N-acetylglucosamine in heart, aorta, brain and skeletal muscle in Brown-Norway rats. Biogerontology 9: 139–151, 2008. doi:10.1007/s10522-007-9123-5. 18185980 PMC2810282

[23] Jones SP, Zachara NE, Ngoh GA, Hill BG, Teshima Y, Bhatnagar A, Hart GW, Marbán E. Cardioprotection by N-acetylglucosamine linkage to cellular proteins. Circulation 117: 1172–1182, 2008. doi:10.1161/CIRCULATIONAHA.107.730515. 18285568

[24] Laczy B, Marsh SA, Brocks CA, Wittmann I, Chatham JC. Inhibition of O-GlcNAcase in perfused rat hearts by NAG-thiazolines at the time of reperfusion is cardioprotective in an O-GlcNAc-dependent manner. Am J Physiol Heart Circ Physiol 299: H1715–H1727, 2010. doi:10.1152/ajpheart.00337.2010. 20833964 PMC2993218

[25] Liu J, Pang Y, Chang T, Bounelis P, Chatham JC, Marchase RB. Increased hexosamine biosynthesis and protein O-GlcNAc levels associated with myocardial protection against calcium paradox and ischemia. J Mol Cell Cardiol 40: 303–312, 2006. doi:10.1016/j.yjmcc.2005.11.003. 16337959

[26] Wang ZV, Deng Y, Gao N, Pedrozo Z, Li DL, Morales CR, Criollo A, Luo X, Tan W, Jiang N, Lehrman MA, Rothermel BA, Lee AH, Lavandero S, Mammen PP, Ferdous A, Gillette TG, Scherer PE, Hill JA. Spliced X-box binding protein 1 couples the unfolded protein response to hexosamine biosynthetic pathway. Cell 156: 1179–1192, 2014. doi:10.1016/j.cell.2014.01.014. 24630721 PMC3959665

[27] Ngoh GA, Facundo HT, Hamid T, Dillmann W, Zachara NE, Jones SP. Unique hexosaminidase reduces metabolic survival signal and sensitizes cardiac myocytes to hypoxia/reoxygenation injury. Circ Res 104: 41–49, 2009. doi:10.1161/CIRCRESAHA.108.189431. 19023128 PMC2712829

[28] Kamemura K, Hayes BK, Comer FI, Hart GW. Dynamic interplay between O-glycosylation and O-phosphorylation of nucleocytoplasmic proteins: alternative glycosylation/phosphorylation of THR-58, a known mutational hot spot of c-Myc in lymphomas, is regulated by mitogens. J Biol Chem 277: 19229–19235, 2002. doi:10.1074/jbc.M201729200. 11904304

[29] Cameron A, Giacomozzi B, Joyce J, Gray A, Graham D, Ousson S, Neny M, Beher D, Carlson G, O’Moore J, Shearman M, Hering H. Generation and characterization of a rabbit monoclonal antibody site-specific for tau O-GlcNAcylated at serine 400. FEBS Lett 587: 3722–3728, 2013. doi:10.1016/j.febslet.2013.09.042. 24113653

[30] Yuzwa SA, Yadav AK, Skorobogatko Y, Clark T, Vosseller K, Vocadlo DJ. Mapping O-GlcNAc modification sites on tau and generation of a site-specific O-GlcNAc tau antibody. Amino Acids 40: 857–868, 2011. doi:10.1007/s00726-010-0705-1. 20706749

[31] Han C, Gu Y, Shan H, Mi W, Sun J, Shi M, Zhang X, Lu X, Han F, Gong Q, Yu W. O-GlcNAcylation of SIRT1 enhances its deacetylase activity and promotes cytoprotection under stress. Nat Commun 8: 1491, 2017. doi:10.1038/s41467-017-01654-6. 29133780 PMC5684413

[32] Muha V, Williamson R, Hills R, McNeilly AD, McWilliams TG, Alonso J, Schimpl M, Leney AC, Heck AJR, Sutherland C, Read KD, McCrimmon RJ, Brooks SP, van Aalten DMF. Loss of CRMP2 O-GlcNAcylation leads to reduced novel object recognition performance in mice. Open Biol 9: 190192, 2019. doi:10.1098/rsob.190192. 31771416 PMC6893399

[33] Thermo Fisher Scientific. Invitrogen™ Click-iT™ O-GlcNAc Enzymatic Labeling System (Online). https://www.fishersci.com/shop/products/molecular-probes-click-it-o-glcnac-enzymatic-labeling-system/C33368 [Last accessed, 27 June 2023].

[34] Wulff-Fuentes E, Berendt RR, Massman L, Danner L, Malard F, Vora J, Kahsay R, Olivier-Van Stichelen S. The human O-GlcNAcome database and meta-analysis. Sci Data 8: 25, 2021. doi:10.1038/s41597-021-00810-4. 33479245 PMC7820439

[35] Marsh SA, Dell'Italia LJ, Chatham JC. Activation of the hexosamine biosynthesis pathway and protein O-GlcNAcylation modulate hypertrophic and cell signaling pathways in cardiomyocytes from diabetic mice. Amino Acids 40: 819–828, 2011. doi:10.1007/s00726-010-0699-8. 20676904 PMC3025273

[36] Brahma MK, Ha CM, Pepin ME, Mia S, Sun Z, Chatham JC, Habegger KM, Abel ED, Paterson AJ, Young ME, Wende AR. Increased glucose availability attenuates myocardial ketone body utilization. J Am Heart Assoc 9: e013039, 2020. doi:10.1161/JAHA.119.013039. 32750298 PMC7792234

[37] Yin X, Cuello F, Mayr U, Hao Z, Hornshaw M, Ehler E, Avkiran M, Mayr M. Proteomics analysis of the cardiac myofilament subproteome reveals dynamic alterations in phosphatase subunit distribution. Mol Cell Proteomics 9: 497–509, 2010. doi:10.1074/mcp.M900275-MCP200. 20037178 PMC2849712

[38] Fahie K, Narayanan B, Zahra F, Reeves R, Fernandes SM, Hart GW, Zachara NE. Detection and analysis of proteins modified by O-linked *N*-acetylglucosamine. Curr Protoc 1: e129, 2021. doi:10.1002/cpz1.129. 34004049 PMC8862748

[39] Thermo Fisher Scientific. Western Blotting Handbook: Techniques and Tools for Publication-Quality Results (Online). https://www.thermofisher.com/us/en/home/global/forms/protein-gel-electrophoresis-western-blotting-handbooks.html.

[40] Ramirez-Correa G, Ma J, Slawson C, Zeidan Q, Lugo-Fagundo NS, Xu M, Shen X, Gao WD, Caceres V, Chakir K, DeVine L, Cole R, Marchionni L, Paolocci N, Hart GW, Murphy AM. Removal of abnormal myofilament O-GlcNAcylation restores Ca^2+^ sensitivity in diabetic cardiac muscle. Diabetes 64: 3573–3587, 2015. doi:10.2337/db14-1107. 26109417 PMC4587639

[41] Ramirez-Correa GA, Jin W, Wang Z, Zhong X, Gao WD, Dias WB, Vecoli C, Hart GW, Murphy AM. O-linked GlcNAc modification of cardiac myofilament proteins: a novel regulator of myocardial contractile function. Circ Res 103: 1354–1358, 2008. doi:10.1161/CIRCRESAHA.108.184978. 18988896 PMC2615199

[42] Zhu C, Guo W. Detection and quantification of the giant protein titin by SDS-agarose gel electrophoresis. MethodsX 4: 320–327, 2017. doi:10.1016/j.mex.2017.09.007. 29872636 PMC5986978

[43] Utter MS, Warren CM, Solaro RJ. Impact of anesthesia and storage on posttranslational modifications of cardiac myofilament proteins. Physiol Rep 3: e12393, 2015. doi:10.14814/phy2.12393. 25952935 PMC4463824

[44] Schneider CA, Rasband WS, Eliceiri KW. NIH Image to ImageJ: 25 years of image analysis. Nat Methods 9: 671–675, 2012. doi:10.1038/nmeth.2089. 22930834 PMC5554542

[45] Gassmann M, Grenacher B, Rohde B, Vogel J. Quantifying Western blots: pitfalls of densitometry. Electrophoresis 30: 1845–1855, 2009. doi:10.1002/elps.200800720. 19517440

[46] Holt GD, Snow CM, Senior A, Haltiwanger RS, Gerace L, Hart GW. Nuclear pore complex glycoproteins contain cytoplasmically disposed O-linked *N*-acetylglucosamine. J Cell Biol 104: 1157–1164, 1987. doi:10.1083/jcb.104.5.1157. 3571327 PMC2114481

[47] Turner JR, Tartakoff AM, Greenspan NS. Cytologic assessment of nuclear and cytoplasmic O-linked *N*-acetylglucosamine distribution by using anti-streptococcal monoclonal antibodies. Proc Natl Acad Sci USA 87: 5608–5612, 1990. doi:10.1073/pnas.87.15.5608. 2116002 PMC54376

[48] Comer FI, Vosseller K, Wells L, Accavitti MA, Hart GW. Characterization of a mouse monoclonal antibody specific for O-linked *N*-acetylglucosamine. Anal Biochem 293: 169–177, 2001. doi:10.1006/abio.2001.5132. 11399029

[49] Teo CF, Ingale S, Wolfert MA, Elsayed GA, Not LG, Chatham JC, Wells L, Boons GJ. Glycopeptide-specific monoclonal antibodies suggest new roles for O-GlcNAc. Nat Chem Biol 6: 338–343, 2010. doi:10.1038/nchembio.338. 20305658 PMC2857662

[50] Kronlage M, Dewenter M, Grosso J, Fleming T, Oehl U, Lehmann LH, Falcão-Pires I, Leite-Moreira AF, Volk N, Gröne HJ, Müller OJ, Sickmann A, Katus HA, Backs J. O-GlcNAcylation of histone deacetylase 4 protects the diabetic heart from failure. Circulation 140: 580–594, 2019. doi:10.1161/CIRCULATIONAHA.117.031942. 31195810

[51] Prakoso D, Lim SY, Erickson JR, Wallace RS, Lees JG, Tate M, Kiriazis H, Donner DG, Henstridge DC, Davey JR, Qian H, Deo M, Parry LJ, Davidoff AJ, Gregorevic P, Chatham JC, De Blasio MJ, Ritchie RH. Fine-tuning the cardiac O-GlcNAcylation regulatory enzymes governs the functional and structural phenotype of the diabetic heart. Cardiovasc Res 118: 212–225, 2022. doi:10.1093/cvr/cvab043. 33576380

[52] Gélinas R, Mailleux F, Dontaine J, Bultot L, Demeulder B, Ginion A, Daskalopoulos EP, Esfahani H, Dubois-Deruy E, Lauzier B, Gauthier C, Olson AK, Bouchard B, Des Rosiers C, Viollet B, Sakamoto K, Balligand JL, Vanoverschelde JL, Beauloye C, Horman S, Bertrand L. AMPK activation counteracts cardiac hypertrophy by reducing O-GlcNAcylation. Nat Commun 9: 374, 2018. doi:10.1038/s41467-017-02795-4. 29371602 PMC5785516

[53] Zhu WZ, El-Nachef D, Yang X, Ledee D, Olson AK. O-GlcNAc transferase promotes compensated cardiac function and protein kinase a o-glcnacylation during early and established pathological hypertrophy from pressure overload. J Am Heart Assoc 8: e011260, 2019. doi:10.1161/JAHA.118.011260. 31131693 PMC6585351

[54] Erickson JR, Pereira L, Wang L, Han G, Ferguson A, Dao K, Copeland RJ, Despa F, Hart GW, Ripplinger CM, Bers DM. Diabetic hyperglycaemia activates CaMKII and arrhythmias by O-linked glycosylation. Nature 502: 372–376, 2013. doi:10.1038/nature12537. 24077098 PMC3801227

[55] Hegyi B, Borst JM, Bailey LRJ, Shen EY, Lucena AJ, Navedo MF, Bossuyt J, Bers DM. Hyperglycemia regulates cardiac K(+) channels via O-GlcNAc-CaMKII and NOX2-ROS-PKC pathways. Basic Res Cardiol 115: 71, 2020. doi:10.1007/s00395-020-00834-8. 33237428 PMC8349245

[56] Dubois-Deruy E, Belliard A, Mulder P, Bouvet M, Smet-Nocca C, Janel S, Lafont F, Beseme O, Amouyel P, Richard V, Pinet F. Interplay between troponin T phosphorylation and O-N-acetylglucosaminylation in ischaemic heart failure. Cardiovasc Res 107: 56–65, 2015. doi:10.1093/cvr/cvv136. 25916824

[57] Watson LJ, Facundo HT, Ngoh GA, Ameen M, Brainard RE, Lemma KM, Long BW, Prabhu SD, Xuan YT, Jones SP. O-linked β-*N*-acetylglucosamine transferase is indispensable in the failing heart. Proc Natl Acad Sci USA 107: 17797–17802, 2010. doi:10.1073/pnas.1001907107. 20876116 PMC2955091

[58] Okuda T. Western blot data using two distinct anti-O-GlcNAc monoclonal antibodies showing unique glycosylation status on cellular proteins under 2-deoxy-d-glucose treatment. Data Brief 10: 449–453, 2017. doi:10.1016/j.dib.2016.12.001. 28054006 PMC5198851

[59] Townsend CL, Laffy JMJ, Wu Y-CB, Silva O'Hare J, Martin V, Kipling D, Fraternali F, Dunn-Walters DK. Significant differences in physicochemical properties of human immunoglobulin kappa and lambda CDR3 regions. Front Immunol 7: 388, 2016. doi:10.3389/fimmu.2016.00388. 27729912 PMC5037968

